# Anti-proliferative effects of qianliening capsules on prostatic hyperplasia *in vitro* and *in vivo*

**DOI:** 10.3892/mmr.2015.3566

**Published:** 2015-03-27

**Authors:** XIAOYONG ZHONG, JIUMAO LIN, JIANHENG ZHOU, WEI XU, ZHENFENG HONG

**Affiliations:** 1 Department of Rehabilitation Medicine, Fujian University of Traditional Chinese Medicine, Fuzhou, Fujian 350108, P.R. China; 2 Department of Fujian Rehabilitation Tech Co-innovation Center, Fujian University of Traditional Chinese Medicine, Fuzhou, Fujian 350108, P.R. China; 3 Department of Academy of Integrative Medicine Biomedical Research Center, Fujian University of Traditional Chinese Medicine, Fuzhou, Fujian 350108, P.R. China; 4 Department of Integrative Medicine, Fujian University of Traditional Chinese Medicine, Fuzhou, Fujian 350108, P.R. China; 5 Department of Pharmacology, Fujian University of Traditional Chinese Medicine, Fuzhou, Fujian 350108, P.R. China

**Keywords:** qianliening capsules, benign prostatic hyperplasia, proliferation, proliferating cell nuclear antigen, cyclin D1, cyclin-dependent kinase 4

## Abstract

Previous studies by our group showed that Qianliening capsules (QC), a clinically proven effective traditional Chinese formulation that has long been used in the treatment of benign prostatic hyperplasia (BPH), is capable of inhibiting BPH *in vivo* and *in vitro* via the promotion of apoptosis, suppression of the EGFR/STAT3 signaling pathway and regulating the expression of sex hormones as well as their receptors. However, the mechanism of its anti-BPH activity has remained to be fully elucidated. The present study aimed to investigate the mechanism underlying the anti-proliferative effect of QC *in vivo* and *in vitro*. Castrated male Sprage-Dawley (SD) rats where subcutaneously injected with testosterone propionate and the WPMY-1 cell line was stimulated with basic fibroblast growth factor in order to generate BPH *in vivo* and *in vitro* separately, both of which were then subjected to QC treatment. Finasteride was used as a positive control drug for the *in vivo* study. In the present study, it was found that treatment with QC or finasteride significantly reduced the prostatic index (PI=prostate wet weight/body weight × 100) in a rat model of BPH (P<0.05). In addition, reverse transcription quantitative polymerase chain reaction (RT-PCR) and western blot analyses showed that QC or finasteride treatment significantly inhibited model construction-induced upregulation of expression of proliferating cell nuclear antigen, cyclin D1 and cyclin-dependent kinase 4 in prostatic tissues of rats with BPH (P<0.05). The *in vitro* study further proved that QC exhibited anti-proliferative properties via G1/S cell cycle arrest in the WPMY-1 cell line, as evidenced by colony formation, flow cytometric cell cycle, immunoblot and RT-PCR analyses. In conclusion, the present study demonstrated that inhibition of cell proliferation via G1/S cell cycle arrest may be one of the underlying mechanisms of the effect of QC on BPH.

## Introduction

Benign prostatic hyperplasia (BPH) is the most common proliferative disease of the prostate in aged males. Symptoms of BPH include urinary urgency, dysuria, nocturia and increased frequency of urination, and, if left untreated, the disease may lead to recurrent urinary tract infections and acute urinary retention, and thereby affect the quality of life (1,2). BPH has been defined as a progressive hyperplasia of glandular and stromal tissues around the urethra. It is characterized by a hyperplastic process predominantly of stromal cells, and was able to be stimulated by local paracrine, autocrine growth factors as well as inflammatory cytokines (3–5). One of these cytokines is basic fibroblast growth factor (bFGF), a member of the family of heparin-binding polypeptide growth factors, which has been implicated in the pathogenesis of BPH by promoting abnormal proliferation of stromal cells (6,7).

Despite the prevalence of BPH, its pathogenesis has remained controversial. The androgenic hormones testosterones and dihydrotestosterone have a significant role in this process, which is at least of permissive nature (8). Growth factors and other hormones including estrogens may also have a function in this process (8). Multiple partially overlapping and complementary theories have been proposed, including embryonic reawakening, stem cell defects, chronic inflammation, imbalance between androgen/estrogen signaling and increased TGF-β signaling, all of which partly explain for the abnormal growth observed in BPH. However, it is now widely accepted that increases in the total number of stromal and epithelial cells, resulting from excessive cell proliferation and/or reduction of cell apoptosis, have a critical role in the development of BPH (9–11).

The division and duplication of all cells, including prostatic stromal and epithelial cells, is regulated by the cell cycle. G1/S transition is one of the two main checkpoints used by cells to regulate the cell cycle progress and thus the cell proliferation. G1/S progression is tightly regulated by cyclin D1 and cyclin-dependent kinase 4 (CDK4) (12,13). PCNA is an acidic nuclear protein that has been recognized as a histological marker for the G1/S phase transition of the cell cycle (14). Therefore, the expression levels of PCNA, CDK4 and cyclin D1 reflect the proliferative state of BPH cells to a certain extent.

To date, no completely effective treatment for BPH has been developed. Besides prostatic surgery, alpha (1)- adrenergic-receptor antagonists [alpha (1) ARAs] and 5 alpha-reductase inhibitors (5ARIs) are two major drug classes which have remained to be used in treating the BPH (15,16). However, all these therapies may have side effects, including orthostatic hypotension, decreased libido and erectile dysfunction. Therefore, a number of herbal medicines that appear to have limited adverse effects have gained increasing popularity in the use for treating BPH (17). Saw palmetto, *Pygeum africanum* and *Ginkgo biloba* leaf extract (18–20) have long been used to treat BPH successfully.

Qianliening capsules (QC) are a widely used Traditional Chinese Medicinal formulation consisting of *Rheum palmatum* L., *Hirudo Medicinalis*, *Astragalus membranaceus* (Fisch.) Bunge, *Achyranthes aspera* and *Cuscuta chinensis* Lam., which has long been used in the clinic and has been shown to be effective in the treatment of BPH (21–24). QC is able to obviously improve a number of lower urinary tract symptoms (LUTS) in BPH patients, including frequency of urination, urinary urgency, thin urine flow and certain other voiding disorders. Previous *in vivo* and *in vitro* studies by our group showed that QC significantly decreased the prostatic volume and weight in BPH model rats via the promotion of apoptosis, suppression of the EGFR/STAT3 signaling pathway and regulation of the expression of sex hormones as well as their receptors (21–24). However, the underlying mechanism of its anti-BPH activity remains to be fully elucidated. Therefore, the present study aimed to evaluate the therapeutic effect of QC on a rat model of BPH, which was generated by castration and subcutaneous injection with testosterone propionate, and the underlying molecular mechanism of the anti-proliferative activity of QC was investigated. In addition, a model of stromal hyperplasia was generated by stimulation of the normal human prostate stromal cell line WPMY-1, a myofibroblast stromal cell line derived from stromal cells of a normal adult prostate, with bFGF, and this *in vitro* model was used to further verify the anti-proliferation mechanism of QC.

## Materials and methods

### Materials and reagents

QC was provided by the Academy of Pharmacology of Fujian University of Traditional Chinese Medicine (Fuzhou, China; FDA approval no. Z09104065). QC was extracted by ultrasonic-assisted extraction. The high-performance liquid chromatography (HPLC) analysis method of QC, which was previously established by our group (25), confirmed its identity and stability according to the drug requirements of China (23). QC were ground into powder, dissolved in distilled water and stored at 4°C. Testosterone propionate injection solution (25 mg/ml) was obtained from the Shanghai General Pharmaceutical Co., Ltd. (batch no. H31020524; Shanghai, China). Fetal bovine serum (FBS), Dulbecco’s Modified Eagle’s Medium (DMEM) and TRIzol reagent were purchased from Invitrogen Life Technologies (Carlsbad, CA, USA). bFGF was obtained from Sigma-Aldrich (St. Louis, MO, USA). SuperScript II reverse transcriptase was provided by Promega (Madison, WI, USA). PCNA (#13110; rabbit monoclonal IgG), cyclin D1 (#2978; rabbit monoclonal IgG), CDK4 (#12790; rabbit monoclonal IgG) and β-actin (#12790; rabbit monoclonal IgG) antibodies as well as horseradish peroxidase (HRP)-conjugated secondary antibodies (#7075) were obtained from Cell Signaling Technologies (Danvers, MA, USA). Cell lysis buffer for western blot analysis, Bicinchoninic Acid Protein Assay kit, SDS-PAGE gel preparation kit, SDS-PAGE electrophoresis buffer, western transfer buffer, polyvinylidene difluoride (PVDF) membrane and Beyo Enhanced Chemiluminescence (ECL) Plus were all obtained from Beyotime Institute of Biotechnology (Shanghai, China). A fluorescein isothiocyanate (FITC)-conjugated annexin V apoptosis detection kit was purchased from BD Biosciences (San Jose, CA, USA). All the other chemicals used, unless otherwise stated, were obtained from Sigma-Aldrich

### Experimental animals

Thirty-two specific pathogen-free grade male adult Sprague-Dawley (SD) rats (200–220 g; 8 weeks old) were purchased from Shanghai Si-Lai-Ke Experimental Animal Ltd. (Shanghai, China). The rats were housed in clean pathogen-free rooms in an environment with controlled temperature (22°C), humidity and a 12-h light/dark cycle with free access to water and a standard laboratory diet. All animal treatments were strictly in accordance with international ethical guidelines and the National Institutes of Health Guide concerning the Care and Use of Laboratory Animals, and the experiments were approved by the Institutional Animal Care and Use Committee of Fujian University of Traditional Chinese Medicine (Fuzhou, China).

### In vivo BPH model construction and drug administration

The testicles of 24 male SD rats were removed under anaesthesia with intraperitoneal phenobarbital (50 mg/kg body weight; New Asia Pharmaceutical Co., Ltd., Shanghai, China). The remaining eight rats were incised above the pelvic region on the ventral side and then sutured without cutting off the testicles, which were allocated as the sham-operated group (Cont). Following castration, the 24 rats were injected subcutaneously into the abdomen with testosterone propionate (5 mg/kg) for 28 consecutive days to induce the BPH, and the rats of the sham-operated group were subcutaneously administered edible oil as a vehicle control. Along with the construction of the BPH model (26,27), the 24 castrated rats were randomly assigned to three experimental groups with eight animals in each: The model group (Model), the finasteride group (Finast; Merck & Co., Inc., Rahway, NJ, USA) and the QC group (QC), which were intragastrically administered with saline (10 ml/kg), finasteride (0.5 mg/kg) or QC (4.5 mg/kg), respectively. After four weeks of treatment, all animals were euthanized using intraperitoneal injection of pentobarbital (100 mg/kg body weight) the prostates from the rats in all groups were removed, weighed and subjected to reverse transcription quantitative polymerase chain reaction (RT-qPCR) assays and western blot analysis.

### Prostatic index (PI)

An analytical balance (ME3002E; Mettler-Toledo International, Inc., Greifensee, Switzerland) was used to measure the prostate weight (PW) and body weight (BW). The prostatic index (PI) was calculated as: PW/BW ×100%.

### Histological examination

The fixed prostatic tissue was dehydrated in a graded ethanol series (Nanjing Chemical Reagent Co., Ltd., Nanjing, China), embedded in paraffin, sliced into serial 5-*µ*m sections, deparaffinized in xylene (Beyotime Institute of Biotechnology), rehydrated in a graded ethanol series and then stained with hematoxylin and eosin (H&E; Beyotime Institute of Biotechnology) for histological observation under a light microscope (BX51T-PHD-J11; Olympus Corporation, Tokyo, Japan).

### Preparation of cell culture

The human prostate stromal cell line WPMY-1 was obtained from the cell bank of the Chinese Academy of Science (Shanghai, China). The cells were grown in DMEM containing 5% (v/v) FBS, 100 Units/ml penicillin and 100 *µ*g/ml streptomycin in a 37°C humidified incubator with 5% CO_2_. The cells were subcultured at 80–90% confluency.

### Evaluation of cell viability by MTT assay

Cell viability was assessed by the MTT colorimetric assay. WPMY-1 cells were seeded into 96-well plates at a density of 1.0×10^4^ cells/well in 0.1 ml medium. Following incubation overnight, the cells were stimulated with bFGF, while unstimulated cells served as a control. The bFGF-stimulated cells were treated with various concentrations of QC (0, 1, 3 or 5 mg/ml) for 24, 48 or 72 h, and unstimulated cells were either left untreated or treated with QC (5 mg/ml) for 24, 48 and 72 h to test the toxicity of QC. At the end of the incubation, 10 *µ*l MTT [Sigma-Aldrich; 5 mg/ml in phosphate-buffered saline (PBS; GE Healthcare Life Sciences, Logan, UT, USA)] was added to each well, and the samples were incubated for an additional 4 h at 37°C. The purple-blue MTT formazan precipitate was dissolved in 100 *µ*l dimethylsulfoxide. The absorbance was measured at 570 nm using an ELISA reader (Model EXL800; BioTek, Winooski, VT, USA).

### Observation of morphological changes

WPMY-1 cells were seeded into six-well plates at a density of 1.0×10^5^ cells/ml in 2 ml medium. Following incubation overnight, the cells were stimulated with bFGF, while unstimulated cells served as a control, and bFGF-stimulated cells were treated with various concentrations of QC (0, 1, 3 or 5 mg/ml) for 24 h. Cell morphology was observed using a phase-contrast microscope (BX51T-PHD-J11; Olympus Corporation), with images captured at a magnification of 20×.

### Colony formation

WPMY-1 cells were seeded into six-well plates at a density of 1×10^5^ cells/well in 2 ml medium. Following incubation overnight, the cells were stimulated with bFGF, while unstimulated cells served as a control, and bFGF-stimulated cells were treated with various concentrations of QC (0, 1, 3 or 5 mg/ml) for 24 h. The cells were then collected and diluted in fresh medium in the absence of QC as well as bFGF and then re-seeded into six-well plates at a density of 1×10^3^ cells/well. Following incubation for eight days in a humidified incubator with 5% CO_2_ at 37°C, the colonies were observed using a phase-contrast microscope at a magnification of 4×, and the colonies consisting of ≥50 cells were counted.

### Cell cycle analysis

Cell cycle analysis was performed by flow cytometry using a FACSCalibur (BD Biosciences) and propidium iodide staining. bFGF-stimulated WPMY-1 cells were treated with or without QC (3 mg/ml) for 24 h, and unstimulated cells were used as a control. All the cells were collected and suspensions were adjusted to a concentration of 1×10^6^ cells/ml, and fixed in 70% ethanol at 4°C overnight. The fixed cells were washed twice with cold PBS and then incubated for 30 min with RNase (8 *µ*g/ml; Sigma-Aldrich) and PI (10 *µ*g/ml; Sigma-Aldrich). The fluorescent signal was detected through the FL2 channel and the proportion of DNA in various phases was analyzed using ModfitLT version 3.0 (Verity Software House, Topsham, ME, USA).

### RNA extraction and RT-qPCR analysis

Total RNA from all samples collected from the *in vivo* and *in vitro* studies was isolated with TRIzol reagent according to the manufacturer’s instructions. Oligo (dT)-primed RNA (1 *µ*g; Shanghai Yingjun Biotechnology Co., Ltd., Shanghai, China) was reverse-transcribed with SuperScript II reverse transcriptase according to the manufacturer’s instructions. The obtained cDNA was used to determine the mRNA levels of PCNA, cyclin D1 or CDK4 by PCR with Taq DNA polymerase (Fermentas; Thermo Fisher Scientific, Waltham, MA, USA). GAPDH or β-actin was used as an internal control. The sequences of the primers used for amplification of PCNA, cyclin D1 and CDK4 in rats are as follows: PCNA forward, 5′-GAC ACA TAC CGC TGC GAT CG-3′ and reverse, 5′-TCA CCA CAG CAT CTC CAA TAT-3′; cyclin D1 forward, 5′-GGA GCA GAA GTG CGA AGA-3′ and reverse, 5′-GGG TGG GTT GGA AAT GAA-3′; CDK4 forward, 5′-CTT CCC GTC AGC ACA GTT C-3′ and reverse, 5′-GGT CAG CAT TTC CAG TAG C-3′; β-actin forward, 5′-ACT GGC ATT GTG ATG GAC TC-3′ and reverse, 5′-CAG CAC TGT GTT GGC ATA GA-3′. The primers used for PCR analysis of the human tissue-derived cell line were as follows: Cyclin D1 forward, 5′-TGG ATG CTG GAG GTC TGC GAG GAA-3′ and reverse, 5′-GGC TTC GAT CTG CTC CTG GCA GGC-3′; CDK4 forward, 5′-CAT GTA GAC CAG GAC CTA AGC-3′ and reverse, 5′-AAC TGG CGC ATC AGA TCC TAG-3′; GADPH forward, 5′-CGA CCA CTT TGT CAA GCT CA-3′ and reverse, 5′-AGG GGT CTA CAT GGC AAC TG-3′. Samples were analyzed by gel electrophoresis (1.5% agarose). Reactions were carried out in a C1000 Thermal Cycler (BioRad Laboratories, Inc., Munich, Germany). The cycling conditions were as follows: Initial denaturation at 95°C for 1 min (1 cycle), denaturation at 94°C for 45 sec and annealing at 5°C below melting temperature for 30 sec (35 cycles), extension at 72°C for 45 sec. A final extension step for 5 min at 72°C was followed by cooling to 4°C. The amplified fragments were analyzed using ethidium bromide stained 1% agarose gels in 1X TBE buffer (all from Beyotime Institute of Biotechnology) at 100 V, until the dye was approximately 75–80% of the way down the gel. The DNA bands were examined using a Gel Documentation System (Gel Doc 2000; Bio-Rad Laboratories, Hercules, CA, USA).

### Western blot analysis

Samples collected from tissues or cells were lysed with cold cell lysis buffer containing phenylmeth-anesulfonylfluoride (Beyotime Institute of Biotechnology) and subjected to SDS-PAGE. The proteins were then electrophoretically transferred onto PVDF membranes, blocked, and then exposed to primary antibodies against PCNA (1:1,000), cyclin D1 (1:1,000) or CDK4 (1:1,000) overnight at 4°C. β-actin (1:1,000) was also measured as an internal control for protein loading. Membranes were then incubated with secondary HRP-conjugated antibodies at 1:2,500 dilution for 2 h at room temperature followed by enhanced chemiluminescence detection.

### Statistical analysis

All values are expressed as the mean of three determinations and data were analyzed using SPSS 16.0 (SPSS, Inc., Chicago, IL, USA). Statistical analysis of the data was performed using Student’s *t*-test and analysis of variance. P<0.05 was considered to indicate a statistically significant difference between values.

## Results

### QC inhibits prostate growth and ameliorates pathological changes of prostate tissue in a rat model of BPH

**Figure 1:**
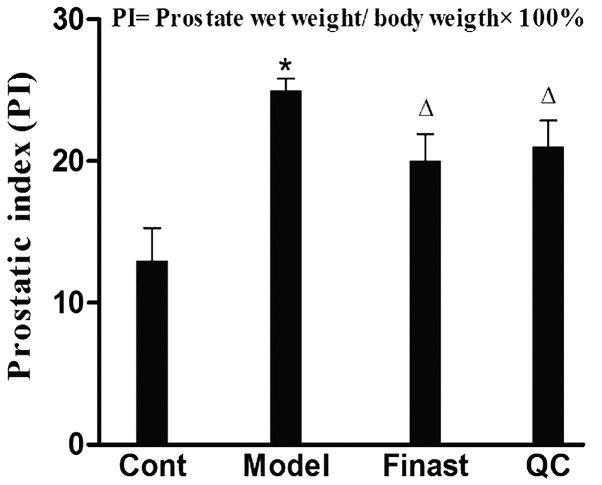
Effect of QC on the PI. Values are expressed as the mean ± standard deviation (error bars) of three experiments. ^*^P<0.05 vs. the control group; ∆P<0.05 vs. model group. QC, qianliening capsules; Cont, control; PI, prostate index; Finast, finasteride.

The *in vivo* therapeutic efficacy of QC against BPH was evaluated by determining the PI in BPH rats. As shown in Fig. 1, the mean PI in the model group was significantly elevated when compared with that in the control group (P<0.05). However, administration of either QC or finasteride significantly reduced the PI in BPH rats (P<0.05), demonstrating the anti-BPH efficacy of QC *in vivo*.

**Figure 2:**
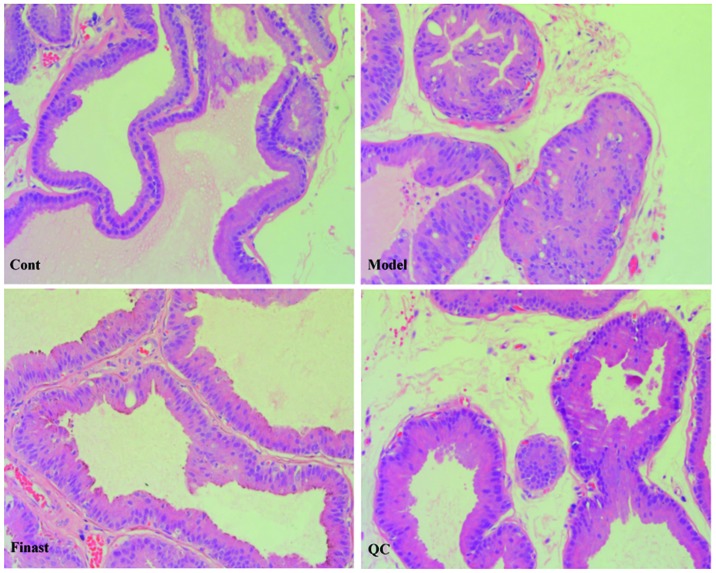
Effects of QC on pathological changes of prostate tissue in BPH rats. Samples were stained with hematoxylin and eosin and observed under a light microscope (magnification, ×100). Cont, control group; Model, model group; Finast, finasteride group; BPH, benign prostatic hyperplasia; QC, qianliening capsules.

Histological changes in prostate tissue of BPH rats were observed using light microscopy following H&E staining. As shown in Fig. 2, low columnar epithelial cells in the control group were arranged as a single-layer secretory lumen that was filled with thin acidophilic materials, whereas the epithelial cells in the model group clearly proliferated to develop excessive glands and cells were arranged as multiple unorganized layers. However, the prostate histopathological damages in BPH rats were significantly ameliorated by QC treatment finasteride (Fig. 2).

### QC treatment inhibits mRNA and protein expression of PCNA, cyclin D1 and CDK4 in prostatic tissue of BPH rats

**Figure 3:**
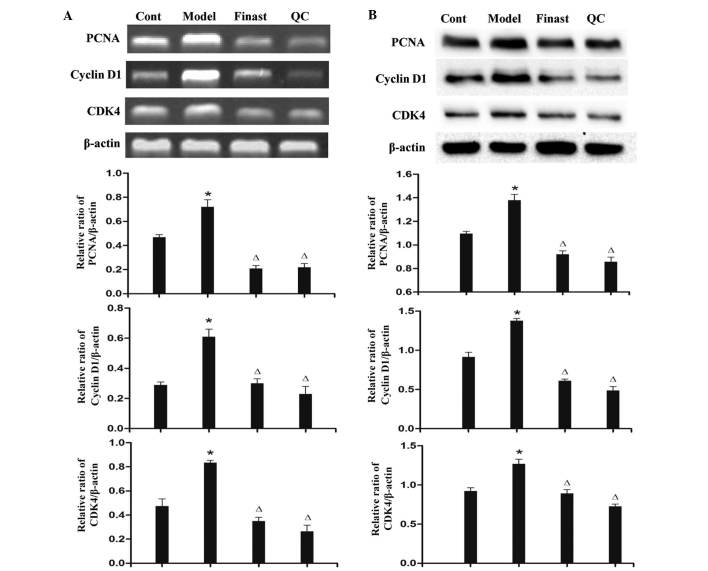
QC treatment inhibited the mRNA and protein expression of PCNA, cyclin D1 and CDK4 in the prostatic tissue of BPH rats. (A) The mRNA levels of PCNA, cyclin D1 and CDK4 in different groups were determined by reverse transcription quantitative polymerase chain reaction. (B) The protein expression levels of PCNA, cyclin D1 and CDK4 were analyzed by western blotting. The results were evaluated by densitometric analysis with β-actin as a loading control. *P<0.05 vs. control group; ^∆^P<0.05 vs. Model group. Cont, control group; Model, model group; Finast, finasteride group; BPH, benign prostatic hyperplasia; QC, qianliening capsules; PCNA, proliferating cell nuclear antigen; CDK4, cyclin-dependent kinase 4.

RT-qPCR and western-blot analysis showed that the mRNA and protein expression levels of PCNA, cyclin D1 and CDK4 in prostatic tissues of the model group were significantly higher than those of the control group (P<0.05) (Fig. 3). Of note, treatment with QC or finasteride profoundly inhibited the protein expression of PCNA, cyclin D1 and CDK4 in the prostatic tissues of BPH rats (P<0.05) (Fig. 3).

### QC suppresses the proliferation ability of bFGF-stimulated WPMY-1 cells

**Figure 4:**
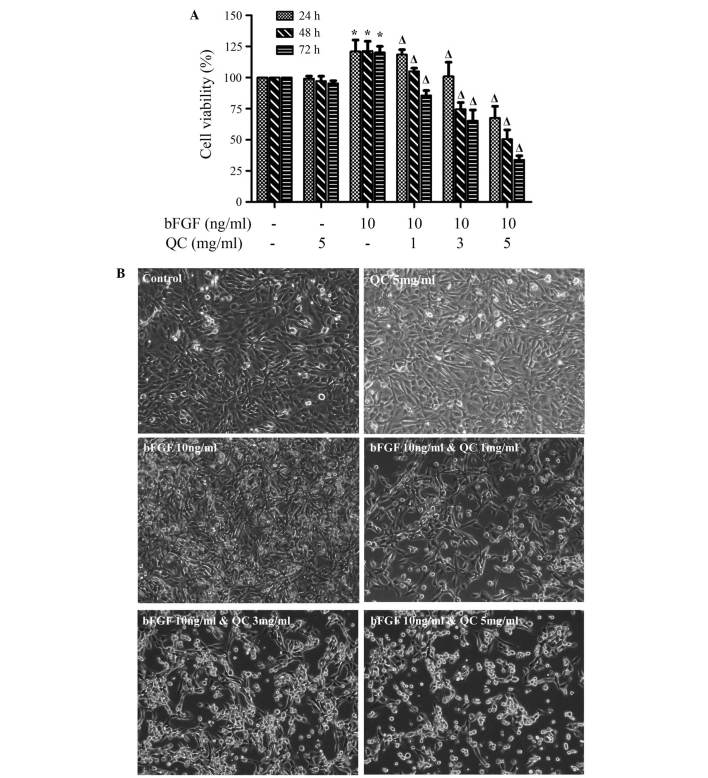
Effect of QC on viability and morphological changes in WPMY-1 cells. (A) MTT assay showed that QC treatment significantly reduced bFGF-stimulated WPMY-1 cell viability in a dose-dependent and time-dependent manner. Data are expressed as the mean ± standard deviation from at least three independent experiments. ^*^P<0.05, compared with the control cells without bFGF stimulation; ^∆^P<0.05, compared with the cells stimulated with bFGF but without QC treatment. (B) Morphologic observation by phase-contrast microscopy (magnification, ×200). WPMY-1 cells without QC treatment showed an excessive cell density and cell number compared to non-bFGF-stimulated WPMY-1 cells, accompanied by morphological changes, including cell shrinkage as well as round and floating cells. bFGF, basic fibroblast growth factor; QC, qianliening capsules.

Histological changes in BPH rats showed that the main effect of dihydrotestosterone was the induction of glandular epithelial hyperplasia in the rat prostate, which was consistent with results from a study by Wang *et al* (28). However, it is well documented that BPH is a proliferative process of the stromal as well as epithelial elements (8–11), and this process was able to be stimulated by local paracrine and autocrine growth factors (3–5). Therefore, in the present study, bFGF was used to stimulate the normal human prostate stromal cell line WPMY-1, a myofibroblast stromal cell line derived from stromal cells of the normal adult prostate, to mimic stromal hyperplasia *in vitro*. The effect of QC on the viability of bFGF-stimulated and non-bFGF-stimulated WPMY-1 cells was determined by MTT and colony formation assays. As shown in Fig. 4A, treatment with 1, 3 and 5 mg/ml QC for different periods of time (24, 48 and 72 h) dose-dependently and time-dependently reduced the bFGF-induced cell viability increase in WPMY-1 cells (P<0.05). However, WPMY-1 cells which were not stimulated with bFGF and treated with 5 mg/ml QC for different periods of time (24, 48 and 72 h) showed no significantly change in viability compared to that of untreated control cells (Fig. 4A). Morphologic observation by phase-contrast microscopy further verified these results. As shown in Fig. 4B, bFGF-stimulated WPMY-1 cells without QC treatment showed an excessive cell density and cell number compared to those of unstimulated WPMY-1 cells. However, treatment with 1, 3 and 5 mg/ml QC for 24 h decreased the cell density and cell number of bFGF-stimulated WPMY-1 cells, accompanied by morphological changes, including cell shrinkage as well as round and floating cells. However, no morphological changes were observed in unstimulated WPMY-1 cells which were treated with 5 mg/ml QC. These results suggested that QC inhibited the growth and viability of bFGF-stimulated WPMY-1 cells in a dose- and time-dependent manner, as described previously (21). Furthermore, QC showed no toxic or proliferative effects on normal WPMY-1 cells which were not stimulated with bFGF.

**Figure 5:**
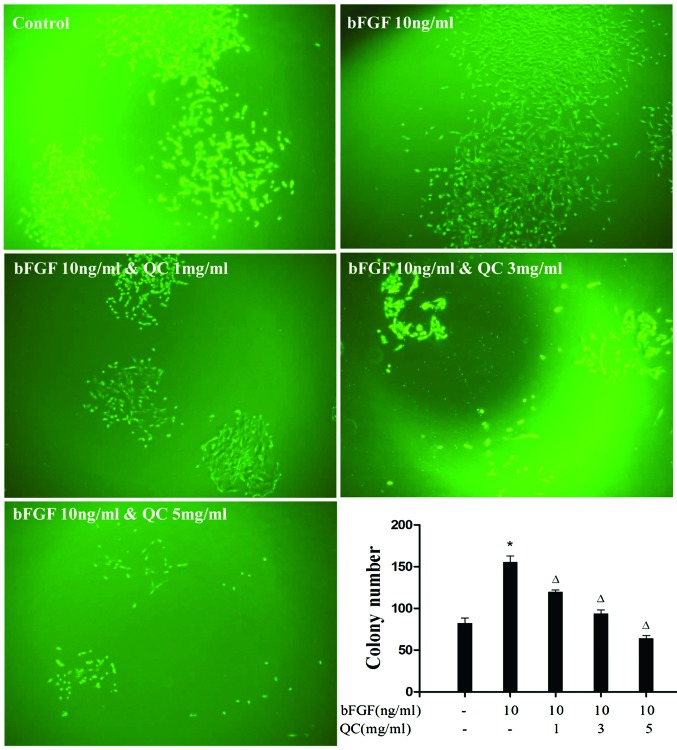
QC suppresses the proliferation ability of bFGF-stimulated WPMY-1 cells. The effect of QC on the proliferation ability of bFGF-stimulated WPMY-1 cells was examined by performing a colony formation assay. Magnification, ×100; a green microscope filter was used. Treatment with 1, 3 and 5 mg/ml of QC for 24 h profoundly suppressed colony numbers (≥50 cells) as well as the colony size in a dose-dependent manner (P<0.05). ^*^P<0.05, compared with the control cells without bFGF stimulation; ^∆^P<0.05, compared with the cells stimulated with bFGF but without QC treatment. Results are presented as the mean ± standard deviation of three independent experiments. bFGF, basic fibroblast growth factor; QC, qianliening capsules.

In addition, the present study examined the effect of QC on the proliferation ability of bFGF-stimulated WPMY-1 cells by performing a colony formation assay. As shown in Fig. 5, treatment with 1, 3 and 5 mg/ml of QC for 24 h profoundly suppressed colony numbers (P<0.05) as well as the colony size in a dose-dependent manner, indicating that QC suppressed the bFGF-induced WPMY-1 cell proliferation.

### QC blocks G1/S progression of bFGF-stimulated WPMY-1 cells

**Figure 6:**
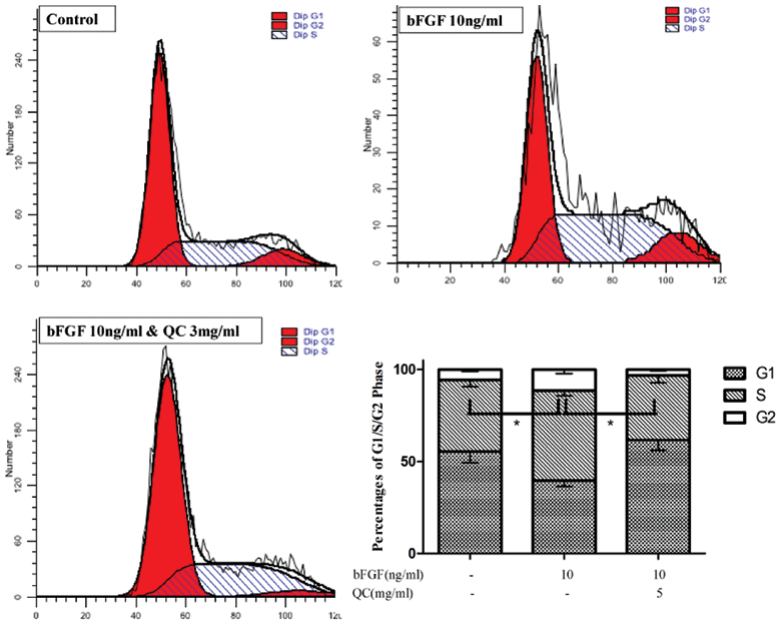
Effect of QC on the cell cycle distribution of WPMY-1 cells. Cells were treated with QC (3 mg/ml) for 24 h, stained with propidium iodide and analyzed by flow cytometry. The proportion of DNA in the G1, S, G2-phase was calculated using ModfitLT version 3.0 software Results are expressed as the mean ± standard deviation of three indepedent experiments, ^*^P<0.05. QC, qianliening capsules.

G1/S transition is one of the two main checkpoints used by cells to regulate cell cycle progression and thus, the cell proliferation. The present study therefore investigated the effect of QC on the G1 to S progression in bFGF-stimulated WPMY-1 cells via propidium iodide staining followed by fluorescence-assisted cell sorting (FACS) analysis. As shown in Fig. 6, the percentage of cells in S-phase following bFGF stimulation was 48.73±2.59%, compared with 38.86±3.68% in the unstimulated group (P<0.05). Furthermore, treatment with 5 mg/ml QC resulted in an S-phase population of 35.23±4.11%, which was significantly different from that in the bFGF-stimulated group without QC treatment (P<0.05). These results indicated that QC inhibits the proliferation of bFGF-induced WPMY-1 cells by blocking the G1 to S-phase transition.

### QC regulates the expression of cyclin D1 and CDK4 in bFGF-stimulated WPMY-1 cells

**Figure 7:**
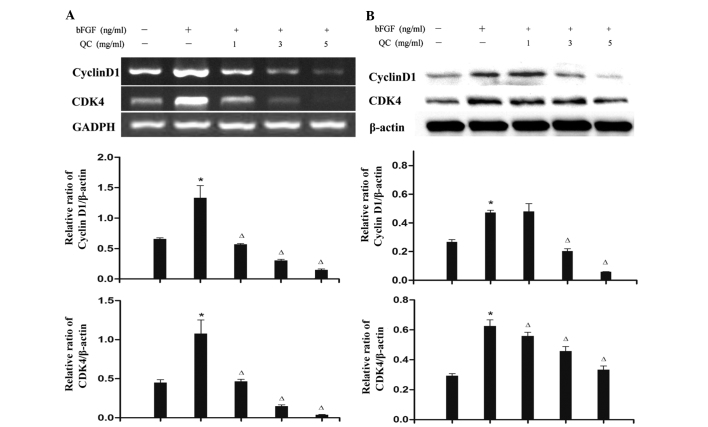
QC treatment inhibited the mRNA and protein expression of cyclin D1 and CDK4 in the WPMY-1 cells. (A) mRNA levels of cyclin D1 and CDK4 in different groups were determined by reverse transcription quantitative polymerase chain reaction. (B) Protein expression levels of cyclin D1 and CDK4 were analyzed by western blotting. The results were evaluated by densitometric analysis with GADPH and β-actin used as loading controls. ^*^P<0.05, compared with the control cells without bFGF stimulation; ^∆^P<0.05, compared with the cells stimulated with bFGF but without QC treatment. Results are expressed as the mean ± standard deviation of three independent experiments. BPH, benign prostatic hyperplasia; QC, qianliening capsules; CDK4, cyclin-dependent kinase 4.

To further verify the mechanism of the anti-proliferative activity of QC, RT-PCR and western blot analyses were performed to examine the mRNA and protein expression levels of cyclin D1 and CDK4 in WPMY-1 cells. As shown in Fig. 7, QC treatment profoundly and dose-dependently reduced the expression of cyclin D1 and CDK4 at the transcriptional as well as the translational level.

## Discussion

As therapeutic approaches for BPH, alpha (1)ARAs, 5ARIs or surgery are widely prescribed to decrease functional or mechanical outlet obstruction. However, as these therapies have side effects, numerous patients seek herbal remedies for BPH, which may generate less negative effects and display therapeutic efficacy. As a traditional Chinese herbal formulation which has long been used in clinical practice, QC has been shown to be effective in the treatment of BPH (21–24). In the *in vivo* experiment of the present study, QC and finasteride significantly reduced the PI, and ameliorated histopathological changes and damage of prostate tissue in BPH rats, which validated the clinical effect of QC. Previous *in vivo* and *in vitro* studies by our group showed that QC exhibited activity against BPH via the promotion of apoptosis, suppression of the EGFR/STAT3 signaling pathway and regulating the expression of sex hormones as well as their receptors (21–24). However, the mechanism of its anti-proliferative activity still remained to be fully elucidated.

BPH is considered to be a proliferative process of the stromal as well as epithelial elements. Cell proliferation is highly regulated by the cell cycle, which consists of the following phases: G1 phase, S phase (DNA synthesis phase) and G2/M phase (mitosis). G1/S transition is one of the two major checkpoints of the cell cycle (29), and is responsible for initiation and completion of DNA replication. G1/S progression is precisely regulated by cyclin D1, which exerts its function via forming an active complex with its CDK major catalytic partners (CDK4/6) (30,31). An unchecked or hyper-activated cyclin D1/CDK4 complex may be responsible for enhanced cellular proliferation and the alteration of cyclin D1/CDK4 complexes is increasingly considered to be a possible target for anti-proliferative therapies (32–34). PCNA is a 36-kD DNA polymerase delta auxiliary protein involved in proliferation and it is specifically expressed in proliferating cell nuclei. PCNA has been recognized as a histological marker for the G1/S phase of the cell cycle (35). In the present study, it was found that the expression of PCNA, cyclin D1 and CDK4 was significantly increased in the BPH model group, which, however, could be significantly inhibited by QC treatment, as evidenced by RT-PCR and western blot analyses. Considering the marked epithelial changes in prostates of BPH rats, the *in vivo* experiment of the present study suggested that QC can inhibit prostate cell proliferation as well as the G1/S transition in prostate epithelial cells of BPH rats by regulating cyclin D1 and CDK4 expression.

However, it is well documented that BPH is considered to be a proliferative process of the stromal and epithelial elements (8–11), and this process was able to be stimulated by local paracrine and autocrine growth factors (3–5). Therefore, bFGF was used in the present study to stimulate the normal human prostate stromal cell line WPMY-1, a myofibroblast stromal cell line derived from stromal cells of normal adult prostate, to mimic stromal hyperplasia *in vitro*. As expected, QC treatment inhibited the proliferation of bFGF-stimulated WPMY-1 cells, which was evaluated by cell viability assay and morphological observation. In addition, a colony formation assay showed that QC had an inhibiting effect on bFGF-induced WPMY-1 cell proliferation. Furthermore, cell cycle analysis showed that QC treatment repressed the G1 to S-phase transition in bFGF-stimulated WPMY-1 cells. Consistent with the inhibitory effect of QC on G1/S transition, RT-qPCR and western blot analyses indicated that QC treatment suppressed the mRNA and protein expression of the G1/S checkpoint proteins cyclin D1 and CDK4 in bFGF-stimulated WPMY-1 cells.

In conclusion, the *in vivo* and *vitro* results of the present study suggested that QC exhibits activity against BPH not only by targeting epithelial cells, but also stromal cells of the prostate. The underlying mechanism of the effect of QC against BPH is the inhibition of cell proliferation by blocking G1 to S-phase transition, which is mediated via suppression of cell cycle checkpoint proteins.

## Abbreviations

QC: Qianliening capsule
BPH: benign prostatic hyperplasia
PI: prostatic index
bFGF: basic fibroblast growth factor
LUTS: lower urinary tract symptoms
alpha (1) ARAs: alpha (1)-adrenergic-receptor antagonists
5ARIs: 5 alpha-reductase inhibitors
DHT: 5-dihydrotestosterone
